# The effects of prognostic nutritional index, systemic immune inflammation index and HALP score on fistula formation, recurrence and mortality in laryngeal cancer patients

**DOI:** 10.1007/s00405-025-09223-0

**Published:** 2025-02-06

**Authors:** B. B. Büyük, F. Toprak, Caner Kılıç, T. Tunçcan, Ceren Öztop

**Affiliations:** Department of Otorhinolaryngology, University of Health Sciences Ankara Dr. Abdurrahman Yurtaslan Oncology Training and Research Hospital, Ankara, Türkiye

**Keywords:** Fistula, Index, Larynx, Recurrence, Survival

## Abstract

**Purpose:**

The aim of this study was to investigate the effects of prognostic nutritional index (PNI), systemic immune inflammation index (SII) and hemoglobin, albumin, lymphocyte, platelet (HALP) score on fistula formation, recurrence and mortality in patients with laryngeal cancer.

**Method:**

The study included 77 patients who underwent total laryngectomy operation between 2018 and 2021. 66 (85.7%) patients underwent primary and 11 (14.3%) patients underwent salvage total laryngectomy. PNI, SII and HALP scores and cutt-off values of all patients were determined and the relationships between pharyngocutaneous fistula (PCF) formation, recurrence and mortality were statistically analysed. The patients with a score less than the cut-off value were divided into two groups as Group 1, and the patients with a score equal to or greater than the cut-off value were divided into two groups as Group 2.

**Results:**

The effect of PNI, SII and HALP on the development of FKF was not significant (*P* = 0.110, *P* = 0.135, *P* = 0.358). The effect of high SII and low HALP score on the development of recurrence was statistically significant (*P* = 0.001, *P* = 0.012). Low PNI increased the development of recurrence, but this increase was not statistically significant (*P* = 0.075). Overall survival rate was 68.8%. The effect of low PNI and HALP on survival was statistically significant (*P* = 0.011, *P* = 0.021). The effect of high SII on survival was not significant (*P* = 0.533).

**Conclusion:**

Low PNI index and HALP score and high SII index are cost-effective simple prognostic biomarkers that are significant in the development of FCF, as well as in the evaluation of recurrence and overall survival in the long-term follow-up of these patients.

## Introduction

Laryngeal cancer is the most common cancer of the respiratory tract after lung cancer and is frequently epithelial in origin. The most common pathological subtype of this cancer is squamous cell carcinoma. Patients frequently present with hoarseness, but symptoms such as dysphagia and neck swelling may also be observed depending on the stage of the disease [1]. In early stages, endolaryngeal surgery or radiotherapy (RT) is performed, while in locally advanced stages (T3), conservative surgical methods such as supracricoid partial surgeries are preferred. However, total laryngectomy becomes the only treatment option in advanced disease (T3, T4a) with high tumour burden, partial surgery or invasion of the trioid cartilage [2].

Total laryngectomy is an operation with difficult preoperative and postoperative management rather than surgery. Preoperative nutritional parameters affect many factors from postoperative recovery to long-term survival. Pharyngocutaneous fistula () is one of the most important complications that delays recovery in the postoperative period and increases the length of hospital stay and thus treatment costs [3]. To date, diabetes mellitus (DM), hypertension (HT), low preoperative hemoglobin and albumin levels have been considered among the known pharyngocutaneous fistula risk factors. In addition, changes in inflammation parameters such as C-reactive protein (CRP) have been reported to be significant in predicting postoperative fistula formation [4].

Long-term recurrence and survival in laryngeal cancers are known to be directly affected by the T and N stages of the disease, and indirectly affected by factors such as age, gender, general performance status, psychology and tumour-related pathological features [5]. Prognostic nutritional index (PNI), systemic immune inflammation index (SII) and hemoglobin, albumin, lymphocyte, platelet (HALP) scores, which are cost-effective biomarkers that can be easily calculated by routine laboratory tests before treatment, are used in calculations of cardiovascular and cerebrovascular events and cancer overall survival [6]. High SII scores and low PNI and HALP scores may have negative effects on complication development, recurrence and survival. These parameters are new biomarkers of systemic inflammation, metabolic impairment and malnutrition and have been investigated in different oncological studies [7].

In this study, we aimed to evaluate the effects of preoperative prognostic nutritional index, systemic immune inflammation index and HALP scores on pharyngocutaneous fistula, recurrence and overall survival.

## Method

The study included 77 patients who underwent total laryngectomy operation due to advanced disease between 2018 and 2021 and whose preoperative laboratory records could be accessed properly and who were N0 because they could affect the study results. Of the patients, 73 (94.8%) were male and 4 (5.2%) were female. Ages ranged between 42 and 81 years (± 8.260) with a mean age of 61.47 years.

Primary laryngectomy was performed in 66 (85.7%) and salvage total laryngectomy in 11 (14.3%) patients. 54 (70.1%) patients were T4, 23 (29.9%) were T3. Small or large pharyngocutaneous fistula (PCF) developed in 17 (22.2%) of the total patients. Patients with DM, post-operative bleeding which may affect the development of PCF were not included in the study. After removal of the main tumour, frozen examination and postoperative specimen reports showed that the surgical margins were tumour-free in all patients. Adjuvant radiotherapy was applied to all patients due to advanced tumour stage.

Routine blood tests (lymphocytes, albumin, neutrophils, platelets and hemoglobin) were used for PNI, SII and HALP score calculations. How to determine these indices and scores is described below [6].

$$\eqalign{{\rm{PNI}}\,{\rm{ = }} & \,\left( {{\rm{10 \times serum albumin }}\left[ {{\rm{g/dL}}} \right]} \right){\rm{ }} \cr & {\rm{ + }}\left( {{\rm{0}}{\rm{.005 \times Lymphocytes/\mu L}}} \right) \cr} $$

$$\eqalign{{\rm{SII = }} & {\rm{Platelet }}\left( {{\rm{109/L}}} \right){\rm{ \times Neutrophil }}\left( {{\rm{109/L}}} \right){\rm{ }} \cr & {\rm{/Lymphocyte }}\left( {{\rm{109/L}}} \right) \cr} $$

$$\eqalign{{\rm{HALP score: }} & {\rm{[hemoglobin }}\left( {{\rm{g/L}}} \right){\rm{ }} \times {\rm{ albumin }}\left( {{\rm{g/L}}} \right){\rm{ }} \cr & \times {\rm{ lymphocytes }}\left( {{\rm{/L}}} \right){\rm{]}}\,{\rm{/ platelets }}\left( {{\rm{/L}}} \right) \cr} $$

Prognostic nutritional index, systemic immune inflammation index and HALP score were calculated and the relationships between fistula formation, recurrence and mortality were analysed statistically. At least 24 months follow-up was used for mortality. Cutt-off values were determined in ROC curve analysis for PNI, SII and HALP scores. Those less than this value were divided into two separate groups as Group 1, and those equal and greater than this value were divided into two separate groups as Group 2.

SPSS 25 statistical packages were used for all analyses. Cox proportional hazards regression was used to analyse the relationship between clinical variables, imaging parameters and pharyngocutaneous fistula formation, recurrence and mortality. ROC curve analysis was performed to calculate the cut-off values of prognostic nutritional index, systemic immune inflammation index and HALP score. These indices and scores were divided into two groups according to cut-off values. F and recurrence effects of the groups were analysed by Chi- square and Fisher analysis. Kaplan-Meier analysis was used for survival analyses. *P* < 0.05 was considered statistically significant.

## Results

Pharyngo-cutaneous fistula developed in 14 (21.8%) patients who underwent primary TL and 3 (17.6%) patients who underwent salvage TL (Table 1). Fistula development according to the type of operation was not statistically significant (*P* = 0.656).

**Table 1:** Main patient and disease characteristics according to tumor site

Characters	*n* (%)
Gender	
• Male	73 (94.8)
• Female	4 (5.2)
T evre	
• T3	23 (29.9)
• T4a	54 (70.1)
Total laryngectomy	77
• Primer	66 (85.7)
• Salvage	11 (14.3)
Pharyngocutaneous fistula	17 (22.2)
• Primer	14 (21.8)
• Salvage	3 (17.6)
Recurrence	
• Yes	9 (11.6)
• No	68 (88.4)
Overall Survival	53 (%68.8)
• Primer	46 (%69.2)
• Salvage	7 (%63.6)

According to the ROC curve, the cut-off values for the development of pharyngo-cutaneous fistula were SII: 797.5 (area 0.653), PNI: 49.21 (area 0.0641), HALP: 42.9 (area 0.611) cut-off values were found. Fistula analyses were performed according to these cut-off values. Accordingly;

PCF developed in 10 (30.3%) of 33 (42.8%) patients in PNI group 1 and in 7 (15.9%) of 44 (57.1%) patients in group 2. The effect of PNI on fistula development was not significant (*P* = 0.110). There was an increase in low PNI scores, but this increase was not statistically significant.

PCF developed in 7 (16.2%) of 43 (55.8%) patients in SII Group 1 and 10 (22.7%) of 34 (44.1%) patients in Group 2. The effect of SII on fistula development was not significant (*P* = 0.135). More fistulae were seen in patients with high scores, but it was not significant.

PCF developed in 10 (25%) of 40 (51.9%) patients in HALP Group 1 and 7 (18.9%) of 37 (49.1%) patients in Group 2. The effect of HALP on fistula development was not significant (*P* = 0.358). It was more common in patients with low score but not significant.

Recurrence developed in 9 (11.6%) patients during follow-up. According to the ROC curve for recurrence, SII: 970 (area 0.858), PNI: 48.6 (area 0.647), HALP: 32.9 (area 0.788) cut-off values were found. The effect of this value on the development of recurrence was analysed. According to this.

Recurrence occurred in 6 (20%) of 30 (38.9%) patients with PNI Group 1 and in 3 (6%) of 47 (61%) patients with PNI Group 2. Low PNI increased the development of recurrence, but this increase was not statistically significant (*P* = 0.075).

Recurrence developed in 2 (3.5%) of 56 (72%) patients in SII Group 1 and 7 (33.3%) of 21 (27.2%) patients in SII Group 2. The effect of SII elevation on the development of recurrence was statistically significant (*P* = 0.001).

Recurrence developed in 7 (24.1%) of 29 (37.6%) patients in HALP Group 1 and in 2 (4%) of 48 (62.3%) patients in HALP Group 2. The effect of lower HALP score on the development of recurrence was statistically significant (*P* = 0.012).

According to survival analysis; 54 (70.1%) of 77 patients were alive. Overall survival rate was 68.8%. Forty-six (69%) of the patients who underwent primary total laryngectomy and 7 (63%) of the patients who underwent salvage laryngectomy were alive, survival was not statistically significant according to operation type (*P* = 0.735).

Fifteen (65.2%) of T3 stage (29.9%) and 38 (70.4%) of T4a stage (70.1%) patients were alive, the effect of T stage on survival was not statistically significant (*P* = 0.116).

Two (22.2%) of 9 (11.6%) patients with recurrence and 51 (75%) of 68 (68%) patients without recurrence were alive, the effect of the presence of recurrence on survival was not statistically significant (*P* = 0.088).

Fifty (68.5%) of 73 (94.8%) male and 3 (75%) of 4 (5.2%) female patients were alive, the effect of gender on survival was not statistically significant (*P* = 0.270).

According to the ROC curve for overall survival, SII: 763 (area 0.547), PNI: 49.3 (area 0.623), HALP: 38.25 (area 0.620) cut-off values were found. Survival analyses were performed according to these values.

**Fig. 1:**
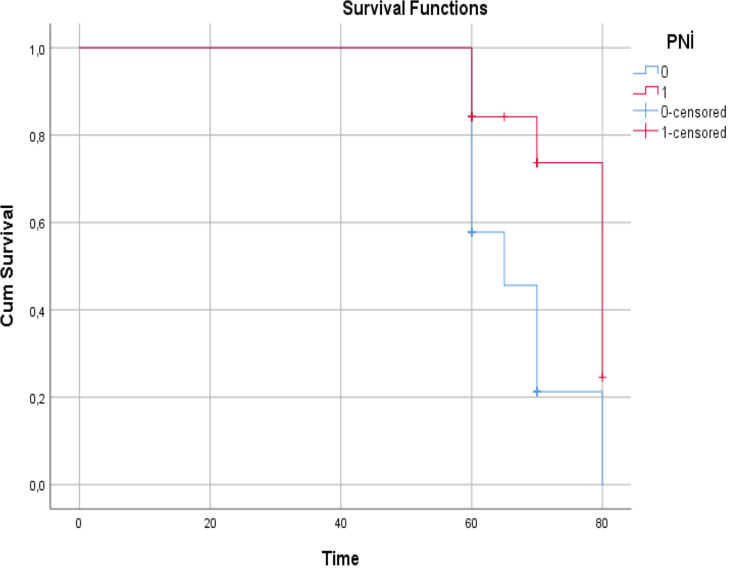
Survival effect of prognostic nutritional index

Nineteen (54.2%) of 35 (45.4%) patients in PNI Group 1 and 36 (85.7%) of 42 (54.6%) patients in PNI Group 2 were alive. The effect of PNI on survival was statistically significant (*P* = 0.011) Fig. 1.

Twenty-five (73.5%) of 34 (44.1%) patients in SII Group 1 and 29 (67.4%) of 43 (55.9%) patients in Group 2 were alive. The effect of SII on survival was not significant (*P* = 0.533) (Table 2).

**Table 2:** Statistical values of index and scores

Characters	PNI (*P* value)	SII (*P* value)	HALP (*P* value)
Pharyngocutaneous fistula	49.21(0.110)	797.5 (0.135)	42.9 (0.358)
Recurrence	48.6 (0.075)	970 (0.001)*	32.9 (0.012)*
Overall Survival	49.3 (0.011)**	763 (0.533)	38.25 (0.021)**

* Chi- Square Fisher’s Exact Test

** Kaplan- Meier

**Fig. 2:**
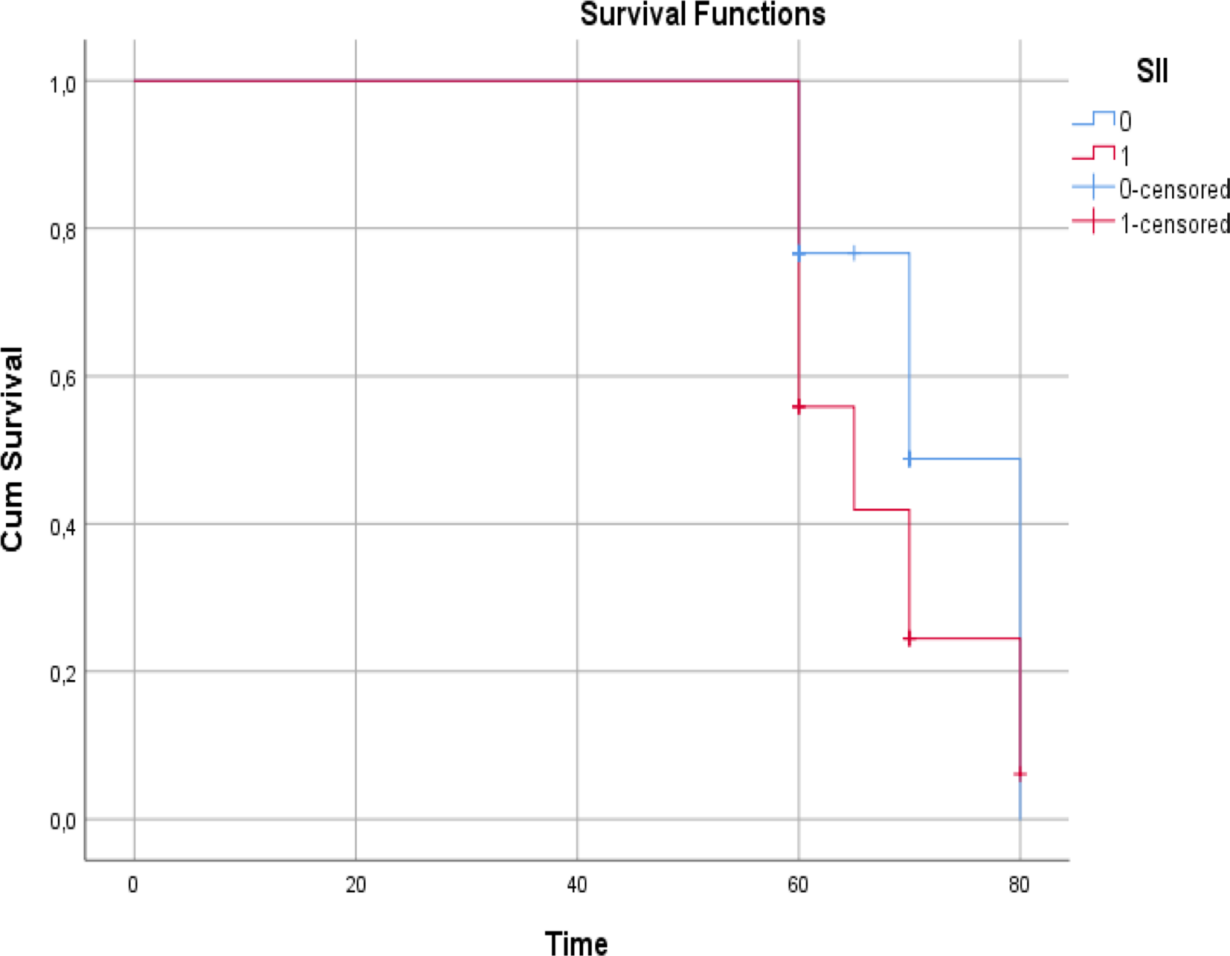
Survival effect of HALP score

Sixteen (50%) of 32 (41.5%) patients in HALP Group 1 and 38 (84.4%) of 45 (58.4%) patients in Group 2 were alive. The effect of lower HALP score on survival was statistically significant (*P* = 0.021) Fig. 2.

## Discussion

Locally advanced laryngeal cancers are known as T3, T4a tumours. In the treatment of these tumours, total laryngectomy, bilateral neck dissection and total thyroidectomy are performed as salvage surgery in patients with high tumour burden, transglottic involvement and T4a tumours, and in patients who have previously received RT and developed recurrence. The most important complication that increases the length of hospital stay and the cost of treatment after surgery is known as FKF. This complication is more common after salvage surgery and is reported to be 13–25% in the literature [8]. In our study, FKF was observed in 22.2% of all patients. It was 21.8% in primary TL patients and 17.6% after salvage surgery. The low rate of fistula after RT may be associated with the low number of operations.

Inflammation is effective as both cause and effect in mucosa-skin/neck/peritoneal fistulas. CRP is a frequently used and economical parameter that shows this inflammation. Its association with different organ anastomotic fistulas has also been reported in the literature [4]. New biomarkers, indices and scores reported in the literature are associated with intestinal fistulas [9, 10]. PNI > 45 has been reported to be a favourable prognostic factor for hepatojejunostomy after liver surgery [11]. Hu et al. reported that preoperative PNI index is a simple and useful biomarker for fistula development after gastrointestinal surgery [12]. Tazeoglu et al. reported that low HALP scores were an effective parameter in studies of fistula development and survival after pancreatic surgery [13].

There are no studies reported with these indices in the literature. In our study, the rate of FKF was found to be increased in low PNI index and HALP scores and high SII index, but this increase was not statistically significant. We think that it will have a significant effect in large series studies.

There are studies in the literature with gastrointestinal region tumours reporting that prognostic nutritional index is a low-cost prognostic biomarker in survival studies [12, 14]. Wu et al. reported that low PNI index was associated with poor prognosis in survival studies including patients with oesophageal cancer [9]. Kazi et al. also reported that an increase in PNI provided absolute benefit to survival in survival studies including patients with colorectal cancer [10]. In another study, PNI and SII indices were reported to be an independent predictor of progression-free survival in patients with advanced breast cancer and they recommended improving the nutritional immune status before treatment [15]. Studies on these indices in head and neck cancers have only recently begun; Momokite et al. studied PNI in patients with end-stage oral cavity cancer and reported that low levels are important markers for survival [16]. Duan et al. also reported that PNI values before radiotherapy are important markers in nasopharyngeal cancer treatment [17].

In our study, although lower PNI index was higher in patients with recurrence, this increase was not significant, and its effect on survival was statistically significant.

The pathophysiology of these markers in the course of the disease; larger tumours and metastatic nodal disease may be accompanied by higher catabolism and cancer-mediated systemic inflammation, which may lead to increased cytokine production, depletion of serum albumin and impaired albumin synthesis in the liver. This may lead to lower PNI levels by increasing the transcapillary transit of albumin [18].

Elevated systemic inflammatory immune response was reported as a poor prognostic marker in a study of gynaecological cancers [19]. Cao et al. also reported that SII may be a promising prognostic marker for recurrence survival in patients with colorectal cancer [20]. Kang et al. also reported that SII is a biomarker associated with lymph node metastasis and prognosis in gastric cancers [21]. In a study conducted with patients with differentiated thyroid cancer, it was reported that patients with high SII had more thyroid cancer than the healthy population and multifocality was increased in these patients [22]. In studies conducted in patients with laryngeal cancer, high SII levels were reported as a poor prognostic marker [23].

In our study, the presence of high SII index was statistically significant for recurrence but not for survival.

Systemic inflammation may affect tumour progression by inhibiting apoptosis, promoting angiogenesis and damaging DNA. Platelets, neutrophils, monocytes and lymphocytes are the four blood components that best represent inflammation in the clinic. Indices calculated with this value are also related to the prognosis of malignant tumours [21].

According to studies with HALP scores, Tarle et al. reported that microvascular flap used in head and neck cancer reconstruction is effective in predicting postoperative complications [24]. In their review, Frag et al. reported that HALP score has a positive prognostic effect in predicting overall survival, progression-free survival, and recurrence-free survival [25]. Sargın et al. reported high HALP score as a favourable prognostic factor in determining survival in gastric cancers [26]. This score has been reported to be an effective biomarker in survival studies in urogenital system cancers [27, 28]. In the head and neck region, low scores were reported to be a significant prognostic biomarker in pharyngeal (nasopharynx, oropharynx, hypopharynx) cancers [29]. In our study, the effect of lower HALP score on both recurrence and overall survival was statistically significant, consistent with the literature.

## Conclusion

We think that prognostic nutritional index, low HALP score and high SII index are effective in the development of pharyngocutaneous fistula, which is one of the most important complications in patients who underwent total laryngectomy with a diagnosis of laryngeal cancer, and that they are cost-effective simple prognostic biomarkers that are significant in the evaluation of recurrence and overall survival in the follow-up of these patients. According to these markers, prediction of the disease course can be made, patient-based treatment strategies (surgical, medical) can be selected and positive contributions can be made to survival times.

## Data Availability

We, as authors, agree to freely make the raw data and materials described in our article available to any scientist who wishes to use them for non-commercial purposes, as long as this does not violate participant confidentiality.
